# Somatic Alterations Impact AR Transcriptional Activity and Efficacy of AR-Targeting Therapies in Prostate Cancer

**DOI:** 10.3390/cancers13163947

**Published:** 2021-08-05

**Authors:** Gaurav Chauhan, Hannelore V. Heemers

**Affiliations:** Department of Cancer Biology, Lerner Research Institute, Cleveland Clinic, NB-40, 9500 Euclid Avenue, Cleveland, OH 44195, USA; CHAUHAG2@ccf.org

**Keywords:** castration, hormonal therapy, biomarker, disease stratification, castration-resistant, castration-sensitive, somatic alterations, genomics, personalized medicine

## Abstract

**Simple Summary:**

For patients whose prostate cancer spreads beyond the confines of the prostate, treatment options continue to increase. However, we are missing the information that is needed to choose for each patient the best treatment at each step of his cancer progression so we can ensure that maximal remissions and prolonged survival are achieved. In this review, we examine whether a better understanding of how the activity of the target for the default first treatment, the androgen receptor, is regulated in prostate cancer tissues can improve prostate cancer treatment plans. We consider the evidence for variability of androgen receptor activity among patients and examine the molecular basis for this variable action. We summarize clinical evidence supporting that information on a prostate cancer’s genomic composition may inform on its level of androgen receptor action, which may facilitate choice for the most effective first-line therapy and ultimately improve prostate cancer treatment plans overall.

**Abstract:**

Inhibiting the activity of the ligand-activated transcription factor androgen receptor (AR) is the default first-line treatment for metastatic prostate cancer (CaP). Androgen deprivation therapy (ADT) induces remissions, however, their duration varies widely among patients. The reason for this heterogeneity is not known. A better understanding of its molecular basis may improve treatment plans and patient survival. AR’s transcriptional activity is regulated in a context-dependent manner and relies on an interplay between its associated transcriptional regulators, DNA recognition motifs, and ligands. Alterations in one or more of these factors induce shifts in the AR cistrome and transcriptional output. Significant variability in AR activity is seen in both castration-sensitive (CS) and castration-resistant CaP (CRPC). Several AR transcriptional regulators undergo somatic alterations that impact their function in clinical CaPs. Some alterations occur in a significant fraction of cases, resulting in CaP subtypes, while others affect only a few percent of CaPs. Evidence is emerging that these alterations may impact the response to CaP treatments such as ADT, radiation therapy, and chemotherapy. Here, we review the contribution of recurring somatic alterations on AR cistrome and transcriptional output and the efficacy of CaP treatments and explore strategies to use these insights to improve treatment plans and outcomes for CaP patients.

## 1. Introduction

Prostate cancer (CaP) remains the most frequently diagnosed non-skin cancer and the second leading cause of cancer-related mortality in American men [1]. When localized, CaP is treated with curative intent via surgical or radiation approaches [2]. The default treatment for non-localized CaP, or CaP that recurs after surgery or radiotherapy, exploits CaP’s well-known dependence on androgens and their cognate receptor, the androgen receptor (AR). Androgen deprivation strategies have been the mainstay for the treatment of metastatic CaP since the early 1940s [3,4,5]. Conversely, the acquired resistance that almost invariably occurs following an initial remission by androgen deprivation therapy (ADT) is a major contributor to the more than 30,000 CaP deaths that occur in the United States each year [4,6,7,8,9].

The concept and scope of ADT, which prevents activation of AR by androgenic ligands, has been reviewed extensively before [4,10]. Over the past decade, a remarkable expansion in the spectrum of ADT drugs and treatments, and in the sequencing and combinations in which these are administered, has occurred. Newer and more potent androgen biosynthesis inhibitors (e.g., abiraterone acetate, usually taken with prednisone) or antiandrogens (e.g., enzalutamide, apalutamide, darolutamide) that were developed as second-line ADT to overcome AR-dependent growth of castration-resistant CaP (CRPC) that emerges after the failure of traditional, first-line ADT drugs, are now increasingly administered earlier in ADT-naïve or castration-sensitive CaP (CS-CaP) [2,11,12]. Throughout this manuscript, we will refer to CS-CaP as CaP that has not yet been exposed to ADT and is ADT-responsive, whereas the term CRPC will be used to designate CaP that has recurred after at least one round of ADT. In the CS-CaP setting, in cases of oligometastatic spread or more widespread dissemination, newer ADT agents are now also more frequently administered in combination with traditional ADT, radiation therapy, or chemotherapy such as docetaxel [13,14,15]. Such combination therapies have shown survival benefit over (first-line) ADT alone [16]. Despite this progress and the benefit from the increase in treatment options, new challenges have occurred. There are for instance concerns that more potent AR inhibition upfront may increase the incidence of neuroendocrine CaP (NEPC), an AR-indifferent, -independent, or even AR-negative CaP phenotype. Such phenotypes occur already in approximately 20% of CRPC cases via lineage plasticity after second-line ADT and are limited in treatment options [8,9,17,18]. The addition of treatment options has augmented also the challenges of how best to design an optimal treatment plan for each patient suffering from metastatic progression and to select a therapy that will yield maximal response for individual patients. Heterogeneity in the duration of remissions following ADT, alone and in combination, has been well-recognized, while objective criteria to predict a patient’s response and thus to select the best treatment strategy for individual patients, are lacking [12,19,20].

A better understanding of the molecular basis for these diverse responses may improve and better inform treatment choices to maximize remissions and prolong survival. Such insights may be derived from taking a closer look at the regulation of AR activity. AR action, which is targeted specifically by ADT, is impacted also by radiation therapy and chemotherapy in CaP cells, and is subject to context- and gene-specific regulation [4,21,22,23]. During CaP progression, some of the protein–protein and protein–DNA interactions that govern this gene selectivity [24] are affected by somatic alterations that show interpatient heterogeneity [25,26]. In this review, we present recent insights into the molecular regulation of AR transcriptional output, the key determinants that contribute to its context-dependent action, and somatic alterations that impact these regulators and occur before any treatment for non-organ confined CaP or are enriched after such treatment during clinical CaP progression. We explore also whether CaP stratification based on such genomic information can improve treatment responses.

## 2. Regulation of AR Activity

### 2.1. AR Structure and Function

**Figure 1:**
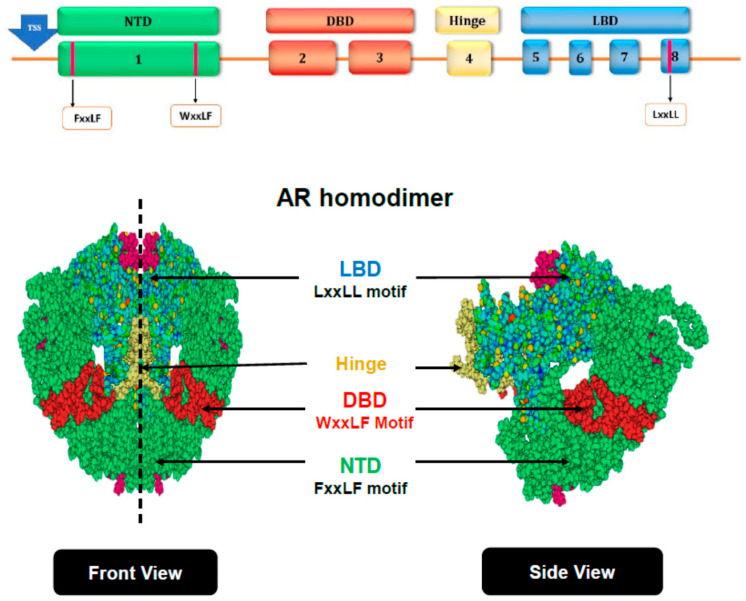
AR structure and function. AR is organized into three major domains: (1) an N-terminal domain (NTD), (2) a DNA binding domain (DBD) that is connected via a small hinge region to (3) a ligand-binding domain (LBD). 1–8 denotes exons. FxxLF, WxxLF, and LxxLL motifs are implicated in AR homodimerization and transcriptional activation. TSS, transcriptional start site (top panel). In full-length AR homodimers, the DBDs and LBDs are located in the core of the dimer and are surrounded by NTDs. Structures generated using cryoEM-derived information in [34] and PyMol (v2.5.0) (bottom panel).

AR is a member of the nuclear receptor family of transcription factors. Its organization in three major domains is similar to that of other NRs and consists of an N-terminal domain (NTD, n-terminal) and a DNA binding domain (DBD, central) that is connected via a small hinge region to a ligand-binding domain (LBD, c-terminal) (Figure 1). Androgen binding to LBD activates AR and causes its re-localization from the cytoplasm to the cell nucleus where AR binds as a dimer to consensus DNA binding motifs known as Androgen Response Elements (AREs) to exert control over transcription of target genes. Transcription is mediated by two distinct activation functions (AFs): one of these, AF2, resides in the LBD and is dependent on ARs ligand-activation; whereas the other, AF1, in the NTD, is constitutively active even in the absence of androgens or the LBD [4,24,27]. In contrast to other NRs, the AF1 in AR is strong, whereas AF2 is weaker. More recently, several so-called AR splice variants have been identified, which lack a functional LBD (and thus AF2) because of AR gene rearrangements or splicing events. The resulting shorter AR forms, which are induced under the selective pressure of ADT, are constitutively active and unresponsive to ADT [28,29]. Although intra- and inter-molecular AR NTD and LBD interactions have been extensively studied and have long been recognized as essential for AR dimerization and ARs transcriptional activity [30,31,32,33], the exact conformation of androgen-activated AR at AREs remains incompletely understood. Because conformational information on the NTD, which is highly unstructured [34,35], has been lacking, until recently any speculations on AR’s potential full-length confirmation had been deduced from microscopy, X-ray crystallography, two hybrid interaction examinations, etc. from one or more individual AR domain(s), and mostly on DBD and LBD [30,32,33]. A recent pioneering cryo-electron microscopy (cryoEM) analysis of androgen-activated DNA-bound AR for the most part confirmed the fragmentary information obtained from these previous efforts and added entirely novel insights in the DNA-binding pattern of full-length AR [36]. The latter study supports a model in which full-length AR dimerizes in a unique head-to-head and tail-to-tail manner, in which the LBD and DBD are located in the center of the AR dimer and tightly dimerize and are surrounded by the NTDs that adopt different modes and conformations of N/C interactions. This structural information was obtained using recombinant AR that was incubated in vitro with relatively small DNA sequences spanning a consensus ARE (32 bp) or a DNA region (324 bp) encompassing AREs found in the enhancer region of the gene-encoding PSA [36]. PSA is a well-studied AR target gene whose serum levels are routinely measured as a surrogate for a patient’s CaP burden [37]. Inclusion of recombinant p300 and SRC-3 protein in cryoEM assays showed that inter- and intra-molecular N/C interactions of AR monomers facilitate also interaction sites for these AR-associated coregulators that are quite different from those found for other NRs [36].

### 2.2. Determinants of AR Transcriptional Output and Target Gene Expression

That AR NTDs can adopt different conformations depending on their interacting protein partners, as suggested by the cryoEM study above, is consistent with previous reports of α-helical sheets forming in the otherwise unstructured AR NTD upon interactions with components of the (general or AR-specific) transcriptional machinery or with ligands [34,35]. Such interactions may enable plasticity in the NTD to adapt different conformations, in which the resulting conformation may be dictated by its specific binding partners [36]. This concept already fits well with allosteric regulation of NR function at DNA-binding sites [24,32,38,39,40,41], but it will be important to validate the overall AR transcriptional complex conformation at endogenously expressed AR target genes.

Supporting the possibility of target gene set-specific AR conformations, multiple literature reports indicate the differential output of AR at distinct targets genes, depending on its interaction with specific ligands, interactors, and DNA. It is well-recognized that the magnitude (fold change) and direction (induced or repressed) of androgen regulation of the hundreds of AR-binding genes that have been discovered to date vary widely. Moreover, the spectrum of AR target genes that are expressed differs between androgen-responsive tissues in general and with the stage of the cell cycle, duration of androgen stimulation, and/or coregulator dependency in CaP cells. For instance, integrated ChIP-Seq and gene expression analysis, after androgen stimulations that varied in duration from 30 min to 24 h, uncovered signatures of AR target genes whose expression was up-or downregulated early (i.e., within 4 h of the start of androgen stimulation) or late (i.e., later than 4 h of the start of androgen stimulation) [42]. These results suggested differences in the molecular machinery in control of these time-sensitive AR target gene signatures. Similarly, a landmark study by the Knudsen lab defined the AR-dependent gene sets and AR cistromes that were common or unique across the different stages of the CaP cell cycle [43]. The authors found that AR binding sites in target genes that show variable expression between stages are enriched also for different transcription factor (TF) binding motifs, which confirms heterogeneity in the molecular regulation of their expression. Moreover, these findings suggested differential involvement in CaP cell biology, which was confirmed by pathway analyses on the cell cycle-specific AR target gene sets. Other non-DNA binding but AR-associated proteins can also influence AR transcriptional output, as shown by work from our group and others that assessed the impact of one or more AR-associated coregulators on the androgen response of AR target gene expression [23,44]. Subsets of AR target genes were impacted by each coregulator, and the resulting coregulator-specific AR target gene signatures were preferentially associated with different aspects of CaP cell biology and clinical progression. Moreover, regions surrounding AR binding sites in these gene sets were also differentially enriched for TF binding motifs in proximity to AREs present in these gene sets. Yet, other studies have revealed an active role for AR-associated TFs over the specific composition of the AR cistrome. For instance, loss of the pioneering transcription factor FoXA1 leads to loss of AR recruitment at sites that are occupied in the presence of FoXA1 and leads to extensive redistribution of AR that binds new sites that are masked in the presence of FoXA1 [45]. To some extent, such reprogramming events occurr also when CaP cells express clinically relevant FoXA1 mutants [46]. Similarly, the transcription factor HoxB13, with important roles in development, is overexpressed in malignant over benign prostate cancer cells, and experimental HoxB13 overexpression induces a malignant AR cistrome in non-malignant epithelial prostate cells [47]. As a ligand-activated TF, AR binds its natural ligands testosterone and DHT but can be bound also by several other compounds including antiandrogens that compete for androgen binding [48]. Some evidence suggests that the specific ligand influences the AR cistrome. The first-generation antiandrogen bicalutamide and the next-generation drug enzalutamide induced genome-wide AR binding patterns, although fewer binding sites were detected when compared to those altered by ligand-activated AR. Moreover, the sites occupied by bicalutamide- or enzalutamide-bound AR did not completely overlap [49]. Ligand-induced specificity of AR for target genes is consistent also with inconsistent effects of molecularly diverse selective androgen receptor modulators (SARMs) on CaP growth [50].

**Figure 2:**
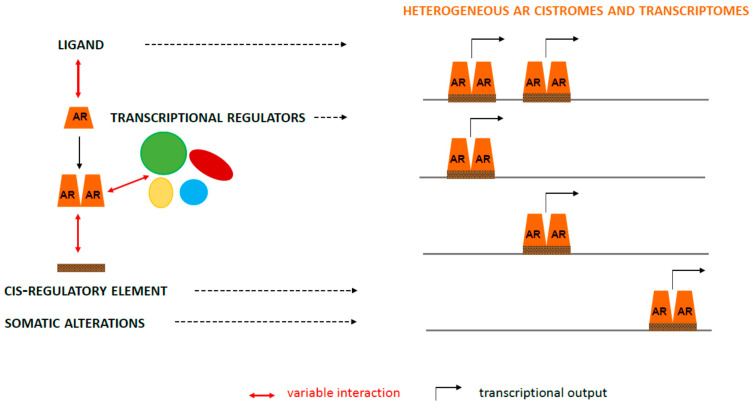
Heterogeneity in AR’s transcriptional output. Variable interactions with ligands (AR monomer), cis-regulatory elements, transcriptional regulators, and somatic alterations (AR dimer) that occur during CaP progression (left panel) lead to markedly different AR cistromes and, ultimately, variability in AR’s transcriptional output (right panel).

Taken together, these observations support that the specific AR DNA-binding motif region, protein interactome, and (in)activating ligand can influence AR target gene transcription (Figure 2). The precise contribution of those regulators of AR action on its transcriptional output remains incompletely understood. Moreover, the implications of (alterations in) these factors for AR-dependent behavior of CaP cells and the success of therapies that impact AR function or innate or acquired resistance to such treatments have not yet fully been explored.

## 3. Heterogeneity in AR Action in Clinical CaP

### 3.1. Differential AR Target Gene Expression and AR Cistromes in Clinical CaP

The use of genome-wide transcriptomics and ChIP-chip, ChIP-Seq, ChIP-Exo or CUT and RUN approaches, by themselves or in an integrated manner, has uncovered a compendia of direct AR target genes, i.e., genes that are androgen-responsive and contain an AR binding site, in CaP cells [49,50,51,52]. Because controlled manipulation of androgen levels in clinical CS-CaP prior to tissue sampling remains difficult, the majority of these studies have been performed on AR-positive CaP cell lines in vitro following exposure to AR agonists or antagonists. However, the behavior of the resulting AR target gene signatures was examined in clinical CaP specimens that were obtained before and after the failure of ADT. Apart from confirming the already well-described shift in AR action during the transition from CS-CaP to CRPC [53,54], these analyses revealed remarkable variability in AR-dependent transcription among patients and even within foci or metastases of the same patient’s CaP [25,55,56]. The diverse interpatient expression has long been recognized for the AR target gene PSA. Multiple groups have reported also similar diversity while examining the expression levels of *bona fide* AR target gene signatures [25,56]. The latter studies mostly summarized overall AR target gene signatures into an activity score, which quantitated expression levels of these genes but did not allow to determine specific contributions of individual genes. To clarify the latter, we recently investigated the expression patterns of 452 AR target genes among 6532 primary CS-CaPs [19]. Unsupervised clustering of CaPs based on AR target gene expression revealed eight distinct AR target gene subsignatures whose differential expression patterns gave rise to five CaP clusters. The individual AR target gene subsignatures differed in their contribution to CaP progression, luminal/basal differentiation, CaP biology, and AR binding site DNA sequences. These data demonstrated that heterogeneity in AR target gene expression among clinical CaPs is due to differential expression of subsets or target genes rather than gradations in the up- or down-regulation of AR transcription overall. These findings were consistent with those from the few studies that defined genomic AR binding sites in steady-state clinical CaP, either before or after ADT [47,54,57]. The latter interrogations noted that the composition of the AR cistromes in both CS-CaP and CRPC specimens is also diverse, with limited overlap in AR binding sites overall at each stage of the disease. The largest AR ChIP-Seq study on CS-CaP tissue to date, which analyzed 88 samples, used a cutoff as low as presence in 25–30% of cases of an AR binding peak to be considered a recurring binding site [57]. Consistent with these findings, we verified that the heterogeneous expression of the 452 AR target genes mentioned above was reflected in the AR binding site patterns using AR-ChIP-Seq data from the same 88 prostate-confined CS-CaPs. We noted that the distribution of AR ChIP-Seq peaks from the 88 CaPs was heterogeneous across the eight gene sets. Moreover, matching RNA-Seq data from the same 88 CaPs showed heterogeneity in AR target gene expression over the eight gene sets, validating that variable gene expression reflects heterogeneity AR cistrome in clinical CaP [19].

### 3.2. Molecular Basis for Differences in AR Action among Clinical CaP

These different lines of investigation independently confirm that variability in AR transcriptional cistromes and transcriptomes exists among clinical CaPs, which raises the question of the molecular basis for such differences. As mentioned above, AR’s ligands, protein interactome, as well as DNA binding sites may all modulate its transactivation function [24]. AR agonists and antagonists are subject to rapid intraCaP metabolism, and the selective pressure of ADT alters expression levels of multiple steroidogenic enzymes [4,58,59]. Such patient-specific conversion events likely impact transcription of AR target genes but are difficult to quantitate routinely in clinical CaP tissue. Regarding the role of genomic AR binding sites in such transcriptional variation, for some time large-scale SNP studies have been indicating the presence of site-specific mutations in AREs [60,61]. More recently, alignment of such sequencing data with CaP cistromes for AR and AR-associated transcriptional regulators (e.g., FoXA1 or HOXB13) of histone marks indicative of transcriptionally (in)active chromatin have confirmed CaP-specific enrichment for mutational events in AR-bindings sites or cis-regulatory elements [62,63]. At least for some (but not all) of these events, massive parallel reporting assays have confirmed that changes in transcription result from these mutated regulatory gene regions [62]. The third modulator of AR’s transcriptional output, its protein interactome, is probably the most extensively studied. Indeed, hundreds of AR-interacting proteins with different cellular functions have been shown to impact and control transactivation by a ligand-activated AR [64,65]. Many of these proteins are differentially expressed during prostate carcinogenesis and CaP progression [27,66]. An example is p300, whose expression is further induced by ADT and chemotherapy in CRPC [67,68]. Somatic alterations have been shown to also impact AR-associated transcriptional regulators. While some occur in larger fractions of clinical CaP cases, e.g., NCOA2 gene amplification in ~20% of CRPC [69], others such as somatic mutations impacting KMT2C or KMT2D genes occur in smaller numbers (~2%) of localized CS-cases [25] but increase in CRPC (~10%). Overexpression of NCOA2 (aka SRC-2), a pivotal regulator of AR transcription, is induced by ADT and causes metastasis and castration recurrence [70], suggesting this overexpresssion facilitates the shift in AR action during CaP progression. Similarly, KMT2C and KMT2D belong to the MLL family of transcriptional coregulators and have been identified as crucial coactivators of AR and potential therapeutic targets in CRPC [71], indicating their altered function in subsets of CaPs may contribute to the observed diverse AR transcriptional outputs.

Since AR-binding ligands or proteins and DNA regions to which AR is recruited contribute to AR transactivation, and at least the concentrations, levels, or sequence integrity of the former two determinants are influenced by AR-targeting therapies, it is tempting to speculate that their status may help classify CaP cases based on AR activity. In this respect, it is important to keep in mind that steroid panels are not routinely determined on clinical CaP tissues, that most somatic alterations events occur at low incidence [72], that coregulators impact AR target gene transcription in a context-dependent manner as a coactivator for some target genes and a corepressor for others [23], and that the majority of such events are not routinely measured in genomic classifiers or targeted sequencing assays that are performed increasingly on clinical CaP to guide treatment decisions. These limitations impede CaP subtyping for AR activity based on such criteria. On the other hand, recent genomic characterization of clinical CaP specimens has led to CaP molecular classifications that may be more amenable. The genomic/transcriptomic marks that are used for subtyping are not present in normal prostate development or benign prostate conditions such as BPH [73,74], but occur in significant subsets of clinical CaP cases, and are routinely determined (e.g., Decipher Biosciences or Foundation One assays) in the course of a patient’s treatment. Noteworthy, the majority of these marks have already been associated with distinct (patterns of) AR activity.

The most frequent somatic alterations in CaP are rearrangements that involve several members of the ETS family of transcription factors such as ERG, ETV1, and ETV4, which have important roles in embryonic development and cell proliferation. The most common of these rearrangements is a fusion between the AR-dependent promoter of the TMPRSS2 gene to the (majority of the) coding region of the gene encoding ERG. This gene fusion, which is present already in ~50% of CS-CaP, results in AR-dependent overexpression of ERG. The TMPRSS2-ERG fusion has been implicated in CaP invasion and migration, and (lack of) treatment response. In view of these findings, inhibitory peptides and peptidomimetics that bind ERG and lead to its proteolytic degradation of the ERG protein have been developed, which attenuate CaP cell invasion and proliferation, and CaP growth [75].

Not surprisingly, information on such alterations has been considered for CaP subtyping. A predominant classification is based on The Cancer Genome Atlas (TCGA) characterization of 333 primary CaPs. Integrated WES, WGS, transcriptomics, and methylation assays on these CaPs led to seven major subtypes encompassing three-quarters of all clinical cases studied. The subtypes were based on the presence of gene fusions involving the ETS transcription family members ERG1, ETV1, ETV4, and Fli, as well as somatic alterations in the E3 ligase SPOP, the pioneering transcription factor FoXA1, and the isocitrate dehydrogenase IDH1 [25]. Six of these seven determinants have been described to influence the transcriptional output of AR.

Indeed, since the first AR ChIP-chip data from promoter arrays on CaP cells in culture, binding sites for ETS factors have been recognized to be enriched in close proximity to AREs [51]. Multiple follow-up studies have confirmed that ETS factors regulate the AR transcriptional activity and that the function of the individual ETS factors and their cistromes in this regard does not completely overlap [76,77]. A study of RNA-seq with ChIP-seq for AR in 88 CS-CaPs and histone marks (H3K27ac, H3K4me3, H3K27me23) in most of these cases uncovered three major subtypes of which two were TMPRSS2-ERG fusion dictated [78]. We have confirmed markedly distinct AR activity scores and AR gene set expressions patterns between CaP clusters based on ERG gene fusion status, in which one cluster was highly enriched (>85% cases positive) whereas another hardly contained any [19]. SPOP mutations that occur in clinical CaP and are used to subtype cases mitigate its E3 ligase function, which prevents degradation of AR and several AR-associated coregulators such as NCOA3 and TRIM24. This may explain increased AR activity scores and differences in AR target gene expression patterns observed in SPOP mutant versus SPOP intact CS-CaPs [79,80]. Similar increased AR activity scores have been noted in CaPs that show point mutations in the AR-associated pioneering factor FoXA1 [25]. While the expression and activity of IDH1, an enzyme that is involved in citrate metabolism, are upregulated by AR [81], reciprocal effects of wild-type or mutant IDH1 on AR activity are less understood. The subclasses that were identified in CS-CaP are present also in metastatic CRPC. Except for SPOP mutations, which are less frequent, and IDH1 alterations, which are near absent in CRPC, the incidence of other molecular marks on which this classification is based are maintained or increased after failure of ADT [25,26].

Further data analyses or deeper sequencing have revealed other clinically relevant sub-classifications, some of which have a bearing on AR transcription output. For instance, a subclass of ERG gene fusion-positive cases frequently showed deletions or mutations in the gene encoding PTEN [25]. Reciprocal feedback between AR and PI3K signaling occurs in PTEN-deficient CaPs [82]. Similarly, about half of SPOP mutant CaPs are characterized by deletions of the chromatin remodelers CHD1 [25], whose experimental loss leads to reorganization of the AR cistrome [83]. About 8% of ADT-naïve CaP and up to 50% of CRPCs show mutations or deletions in the gene encoding p53 that abrogates wild-type p53 function [25,84]. Deletion of p53 function is associated with changes in the composition of the AR cistrome in cultured CaP cells [85]. Our AR target gene pattern analyses on thousands of clinical CaP cases confirmed the preferential association of target gene set expression and presence of somatic alterations in the CHD1, PTEN, and p53 genes, supporting their link with AR transactivation [19].

It should be noted that alternative CaP classification methods have been proposed in which subclasses also vary in AR activity. As an example, the PAM50 classifier, which is now widely used to subtype breast cancer into molecular classes with varying prognoses, has been applied to localized CS-CaP [86]. Three CaP PAM50 subclasses, namely luminal A, luminal B, and basal subtypes in CaP, have been recognized. We noted differences in the expression of AR and AR target genes between PAM50 subclasses, with PAM50 basal CaPs showing lower AR activity scores but higher expression of AR target gene sets that are enriched in roles in organ and system development [19].

## 4. AR Target Gene Expression, Associated Genomic Marks, and CaP Treatment Responses

The fact that genomic alterations can impact CaP aggressiveness, progression, and treatment response, has long been recognized. For instance, localized CS-CaP with a higher number of copy number alterations have a shorter time to biochemical relapse, the most unfavorable prognosis, and more frequently metastasize after surgery [69,87]. Because of these observations, it has been proposed that considering the overall level of such alterations and a better understanding of the specific alterations that contribute most to the unfavorable CaP progression may help distinguish low-high risk primary CS-CaP, serve as prognosticator, or assist in deciding the aggressiveness of a treatment course. The frequency of somatic alterations is markedly increased in CRPC [26], where they are routinely measured in tissue biopsies or in circulating tumor DNA.

In this regard, it is important to note that several of the genomic markers or distinct gene expression patterns used to subtype CaP have been associated with CaP progression and response to CaP treatments that directly target AR or are impacted by AR action. Indeed, although ADT drugs were designed to specifically prevent AR action, other treatment options that are currently considered for first-line treatment of metastatic CaP and were not developed as AR inhibition, also either target aspects of AR function or are impacted by AR inhibition. The best-recognized example is synergistic effects by combining ADT and radiation therapy to control disease progression in localized CS-CaP. The molecular basis for this clinical benefit has been AR’s control over a subset of AR target genes that are induced by DNA damage and mediate DNA repair. ADT thus prevents the resolution of double-strand breaks and resistance to DNA damage, enhancing the impact of radiation therapy [21,88]. The extent to which such cross-talk applies also to commonly used CaP chemotherapeutics such as docetaxel for cabazitaxel is not entirely clear. However, some evidence supports that the expression of AR target genes may be differentially affected by docetaxel. For instance, PSMA levels were not affected, yet AR and PSA expression were down-regulated in a dose-dependent manner, and overexpression of AR partially abrogated the cytotoxic effects of docetaxel [22]. In addition, although not routinely used to treat CaP, doxorubicin inhibited the expression of genes with consensus androgen response elements (cAREs) that drive proliferation but not genes with selective elements (sAREs) that promote differentiation [89].

Whether this implies that genomic marks that are increasingly determined in a patient’s CaP and are linked to AR action can also predict treatment response to AR-targeting of impacting therapies is not yet entirely clear, although evidence is emerging that this may be the case. For instance, SPOP mutations were associated with higher activity of an AR target gene signature [25]. SPOP alterations drive prostate tumorigenesis in vivo, increase proliferation, and are a transcriptional signature for human CaP in mouse prostate organoids [90]. Nonetheless, analysis of a gene expression signature that predicts the SPOP mutant subclass in CS-CaP tissues revealed that the SPOP mutant subclass associates with a lower frequency of positive margins, extraprostatic extension, and seminal vesicle invasion at the time of surgery. In addition, SPOP mutant CaPs were associated with a favorable prognosis with improved metastasis-free survival, particularly in patients with high-risk preoperative PSA levels [91]. Similarly, in a CRPC patient cohort, SPOP mutations and/or CHD1 loss was associated with a higher response rate to the ADT drug abiraterone and a longer time to progress on abiraterone, supporting the theory that this subtype of CaPs may be highly sensitive to ADT [92]. A recent study isolated CHD1 deletion as a subtype-specific late progression event in SPOP mutant CS-CaPs. SPOP mutant only and SPOP mutant/CHD1-deleted CS-CaPs shared early tumorigenesis but distinct pathways toward progression, and the cases with combined SPOP/CHD1 alterations were associated with worse prognosis [93].

ERG gene fusions represent the most frequent somatic alteration and are present already in half of localized CS-CaP [25,26], yet their relevance to CaP progression, treatment response, and the outcome remains incompletely understood. ERG expression, a surrogate for the presence of ERG fusion proteins, does not appear to be a useful biomarker in predicting response to ADT in patients with high-risk prostate cancer [94], nor does ERG gene fusion status in CRPC inform on response to abiraterone acetate [95]. Although preclinical data suggest that TMPRSS2-ERG gene fusions may be a surrogate for DNA repair status and therefore a biomarker for DNA-damaging agents such as radiotherapy [96], in two image-guided radiotherapy cohorts, TMPRSS2-ERG status was not prognostic for biochemical recurrence-free survival [97]. These findings indicated that ERG gene fusion status is unlikely to be a predictive factor for radiation response. However, in patients suffering from metastatic CS-CaP treated with docetaxel in addition to ADT, expression of ERG was associated with favorable relapse-free survival, suggesting ERG expression may have a potential predictive value with respect to the effectiveness and outcome of docetaxel chemotherapy combined with ADT [98].

A subclass of ERG+ cases is enriched for deletion of wild-type PTEN function, and PTEN deletions in ERG+ cases reflect a late event subtype with more aggressive features such as increased locoregional stage compared to ERG fusion-positive and PTEN deletion-negative cases [25,93]. Loss of wild-type PTEN function by itself is well recognized to convey aggressive CaP behavior. In genetically engineered mouse models, prostate-specific PTEN loss induced rapid CaP initiation and aggressive progression and rendered ADT less effective [99,100,101,102]. Several independent studies have also shown that PTEN loss confers a worse prognosis. CS-CaP patients undergoing brachytherapy whose cancer showed concurrent ERG rearrangement; PTEN deletion demonstrated significantly worse relapse-free survival rates compared with those with ERG or PTEN wild-type; combined ERG rearranged; and PTEN deletion was independently associated with biochemical recurrence [103]. In analyses of post-surgery outcomes of CS-CaP patients enrolled in clinical trials of intense neoadjuvant ADT prior to surgery, PTEN loss was also associated with biochemical recurrence and CaP ERG positivity, and PTEN losses were associated with more extensive residual tumors [104,105]. In another study, targeted biopsies from men with intermediate- to high-risk CS-CaP before receiving 6 months of ADT plus enzalutamide were used for whole-exome sequencing and immunohistochemistry (IHC); loss of chromosome 10q (containing PTEN) and alterations to p53 were predictive of poor response, as were the expression of nuclear ERG on IHC [106].

While FoXA1 mutant CS-CaPs show higher AR activity [25], the link between such mutations and CaP outcome and especially treatment response is not as well studied. A recent investigation used an RNA signature to assess mutant FOXA1 status and found that CS-CaPs predicted to be FOXA1 mutant were significantly associated with higher Gleason scores, shorter time to biochemical recurrence, and more rapid progression to metastatic disease than unaltered cases [46].

The studies above focus on first-fine treatments for CS-CaP. Metastatic CRPCs that have already acquired resistance to at least one line of treatment were also characterized genomically. Evaluating the genomic and transcriptomics data from CRPC tissues and circulating tumor DNA from CRPC patients for their impact of features on clinical outcome and specifically treatment response, it was shown that loss of wild-type p53 function was associated with a shorter time of treatment with second-generation antiandrogens. These findings established several of the genomic marks used to subtype CaP as drivers of resistance to first-line AR-directed therapy [84,107].

Moreover, CaP classes derived from methods other than the TCGA-based subtyping have also been suggested to display variable treatment responses. For instance, luminal- and basal-like PAM50 CS-CaPs demonstrate a difference in clinical behavior, in which luminal B CaPs respond better to postoperative ADT than do patients with non-luminal B tumors [86]. These results suggest again that genomics/transcriptomics marks could be used to personalize CaP treatments by predicting which men may benefit most from ADT after surgery.

To ascertain if the information on such marks may facilitate better and informed choices between the multiple treatments or decisions on whether or not to combine such treatments for first-line CS-CaP treatment, we expanded our analyses of AR target gene expression patterns on 6532 CS-CaP clusters [19]. To this end, we aligned the CaP clusters that were generated based on differential expression patterns for eight AR target sub-signatures with gene signatures designed to predict response to ADT [108], radiation therapies [109], and chemotherapeutics such as docetaxel or dasatinib [110]. To derive further insights, we also included scores for gene signatures that reflect the PAM50 classifier basal, luminal A, and luminal B subtypes [86], and compared the behavior of the 452 AR target genes in the CaP clusters to that of AR activity scores that have been used by other groups to assess AR activity in clinical CaP [25,56]. These comparisons provided novel insights into the CaP behavior in terms of AR activity, CaP basal/luminal differentiation, and treatment responses. For instance, the cluster that contained CaPs with the highest basal PAM50 score showed also the lowest AR activity as measured by AR activity score, the lowest predicted response to ADT and docetaxel, but the highest predicted response to radiation therapy. Moreover, this cluster also harbored a subcluster that was most responsive to multikinase dasatinib, which inhibits also SRC that selectively impacts AR target gene expression [111]. While this CaP cluster had the lowest AR activity score, AR target genes that show high expression in this cluster were specifically involved in organ and system development, had AR binding sites that were enriched for binding motifs for transcription factors that are relevant to such developmental processes and embryogenesis and stemness, and tended to be retained in CRPC. In contrast, another cluster showed almost exclusively luminal (and mostly luminal B) PAM50 features, which were accompanied by the highest predicted response to ADT and docetaxel but the lowest response to radiation therapy. The latter features were associated with upregulation of expression of other subsets of AR target genes that displayed none of the developmental features. The remaining three clusters again showed different combinations of target gene set expression, predicted responses to treatment, and levels of basal/luminal differentiation [19].

## 5. Future Directions

Taken together, the studies summarized above support marked differences in AR transcriptional output in clinical CaP. Evidence is emerging that somatic alterations that are linked to fractions of AR action and are routinely assessed in the course of a patient’s treatment course may be associated with preferential response to ADT, radiation therapy, and/or chemotherapy (Table 1). Although these findings are promising and may have far-fetching implications to improve patient care and to move forward in the clinic, several outstanding questions and limitations remain that will need to be addressed. For instance, the majority of findings linking subtypes with treatment responses and CaP outcomes have been derived from a single institution or one database only, mostly at a single stage of CaP progression, and have yet to be validated in clinical trials or tested experimentally in preclinical animal studies. Other caveats relate to the extent to which intrapatient CaP heterogeneity may impact CS-CaP and CRPC tissue sampling [55,56] and thus a reliable readout for the genomic mark of interest, or whether all point mutations or amplications impacting a “classifying gene” have the same biological consequences. In our analyses, the AR target gene patterns we observed between CaP clusters were preferentially associated with CaP molecular subtypes, differentiation, and predictors of treatment response, rather than with clinical variables routinely used for CaP prognosis such as serum PSA levels, Gleason scores, and CaP pathological stages [19]. This is consistent with the previous lack of success in using clinical parameters to foresee the outcome of CaP treatments. Pretreatment serum levels of testosterone or adrenal androgens were considered, but were modestly predictive of response to ADT [112,113,114,115]. At the molecular level, ADT response was associated with AR (variant) expression levels [116], length of AR CAG repeats that affect activity of AR [117,118], expression of steroidogenic enzymes targeted by ADT such as CYP17A1 [119], and germline polymorphisms in androgen transporters [120]. Expression status of two AR target genes, PSA and PSMA, in CaP circulating tumor cells, were used as a measure of AR activity and correlated with CaP response to ADT [121]. These data indicate that a reliable readout of AR’s transcriptional output in CaP specimens may be valuable in predicting (non-)response to treatments, in this case, ADT.

Evaluating genomic subtypes may eventually outperform clinical parameters previously used or currently considered as predictive biomarkers. However, it is important to consider also that about a quarter of CS-CaP cases already do not fall in any of the seven TCGA subclasses identified [25], suggesting other markers may be needed or useful. In a prospective study of patients with metastatic CRPC treated with first-line abiraterone, an RNA-based signature derived from circulating tumor cells, pretreatment expression of HOXB13, which controls a malignant AR cistrome [47], identified patients with poor overall survival, with a subset also expressing the ARV7 splice variant, a version of AR lacking a function LBD [122]. Regarding the latter, amplification, gene rearrangements, and splicing events occur in the AR gene locus under the pressure of ADT. Because they can attenuate the therapeutic efficacy of second-line ADT, they have been proposed as predictive biomarkers to such treatments [12]. Since we focused on the regulation of AR transcriptional output, in this review we have not covered such alterations at the AR gene. Another consideration is whether somatic DNA and RNA readouts provide comprehensive information regarding treatment efficacy. For instance, the germline status of genes relevant to AR action in CaP may also be useful to assess, as exemplified by the gene encoding the steroidogenic enzyme HSD3B1 for which the adrenal-permissive allele inheritance that allows for rapid dihydrotestosterone synthesis from adrenal androgen precursors confers worse outcomes and shorter survival after castration in low-volume CaP and poor outcomes after abiraterone or enzalutamide treatment for CRPC [123]. It stands to reason that the protein level may provide additional, as yet unappreciated, levels of relevant information, raising the question of the scope, platform, and type of biomarker assay(s) that will prove most useful to ascertain biomarker information. A related question pertains to the manner in which CaP tissues and/or cells for analysis of genetic alterations are obtained, i.e., via an invasive versus non-invasive method. In respect to the latter, liquid biopies have been gaining traction as they are minimally invasive; allow for early detection of CaP genomic make up, markers of recurrence, treatment response, or CaP progression; and if needed allow for repeated measurements of genomic marks on circulating tumor cells or circulating tumor DNA during a patient’s treatment plan [124,125]. Nonetheless, collectively, the findings suggest further examination is warranted and may lead to much-needed biomarkers to guide systemic treatment decisions in non-organ-confined CaP based on the activity of AR, the target for default first-line therapies.

## Figures and Tables

**Table 1:** Somatic alterations that have been linked to differential AR action may be associated with diverse responses to CaP treatments.

Classification	Class	Variable AR Action?	Variable CaP Outcome and Treatment Response?	References
**TCGA**	ERG	Yes	Yes, predicted and clinical	[19,25,98,106]
	ETV1	Yes		[19,25]
	ETV4	Yes		[19,25]
	Fli	Yes		[19,25]
	SPOP	Yes	Yes, predicted and clinical	[19,25,91,92,93]
	FOXA	Yes	Yes, clinical	[25,46]
	IDH1	Yes		[25]
**PAM50**	Basal	Yes	Yes, clinical	[19,86]
	Luminal A	Yes	Yes, clinical	[19,86]
	Luminal B	Yes	Yes, clinical	[19,86]
